# Speeding up the brain: when spatial facilitation translates into latency shortening

**DOI:** 10.3389/fnhum.2012.00330

**Published:** 2012-12-19

**Authors:** Anne-Lise Paradis, Shasha Morel, Peggy Seriès, Jean Lorenceau

**Affiliations:** ^1^UPMC Univ Paris 06, UMR-S975 UMR 7225, Centre de Recherche en NeuroscienceEquipe Cogimage, Paris, France; ^2^Inserm U 975, Centre de Recherche en NeuroscienceEquipe Cogimage, Paris, France; ^3^CNRS UMR 7225, Centre de Recherche en NeuroscienceEquipe Cogimage, Paris, France; ^4^ICMEquipe Cogimage, Paris, France; ^5^Laboratoire de Psychopathologie et Neuropsychologie, Université Paris 8Saint-Denis, France; ^6^Centre de NeuroImagerie de Recherche - CENIR, CRICM, UPMC/Inserm UMR-975, CNRS 7225, Hôpital Pitié-SalpêtrièreParis, France; ^7^School of Informatics, Institute for Adaptive and Neural Computation, The University of EdinburghEdinburgh, UK

**Keywords:** horizontal connections, magnetoencephalography (MEG), contrast, contour detection, cortical latencies, neural facilitation, activity propagation

## Abstract

Waves of activity following a focal stimulation are reliably observed to spread across the cortical tissue. The origin of these waves remains unclear and the underlying mechanisms and function are still debated. In this study, we ask whether waves of activity modulate the magnetoencephalography (MEG) signals recorded in humans during visual stimulation with Gabor patches sequentially flashed along a vertical path, eliciting a perception of vertical apparent motion. Building upon the functional properties of long-rang horizontal connections, proposed to contribute to spreading activity, we specifically probe the amplitude and latency of MEG responses as a function of Gabor contrast and orientation. The results indicate that in the left hemisphere the response amplitude is enhanced and the half height response latency is shortened for co-aligned Gabor as compared to misaligned Gabor patches at a low but not at a high contrast. Building upon these findings, we develop a biologically plausible computational model that performs a “spike time alignment” of the responses to elongated contours with varying contrast, endowing them with a phase advance relative to misaligned contours.

## Introduction

Recent optical imaging and electrophysiological recordings in cat and monkey reported waves of activity propagating slowly across the visual cortex after a focal visual stimulation (Grinvald et al., 1994; Bringuier et al., 1999; Jancke et al., 2004; Benucci et al., 2007; Nauhaus et al., 2008, 2012; Meirovithz et al., 2010; Chavane et al., 2011). The distance travelled and the speed of these waves depend on contrast, with lower contrast eliciting slower waves propagating over larger distances (Nauhaus et al., 2009; Meirovithz et al., 2010). Whether these waves play a role in visual processing remains unclear and the underlying mechanisms are still debated (Ray and Maunsell, 2011; Nauhaus et al., 2012). In cortical space, traveling speeds are relatively slow (0.1–1 m/s, Grinvald et al., 1994; Jancke et al., 2004; Benucci et al., 2007; Sharon et al., 2007). These speeds are commensurable with the estimates of propagation speeds through thin unmyelinated long-range axons running parallel to the cortical surface (Bringuier et al., 1999; Bullier, 2001; Hupé et al., 2001a), suggesting that long-range lateral connections contribute to wave propagation (Bringuier et al., 1999; Benucci et al., 2007; Nauhaus et al., 2009, 2012). If true, the functional architecture of lateral connections could constrain the wave dynamics and provide hints on their functional role. Key aspects to take into consideration are the speed of propagation through horizontal long-range connections as well as orientation and contrast that shape the responses of neurons linked by lateral connections.

Extensive electrophysiological (Kapadia et al., 1995, 1999, 2000; Polat et al., 1998), anatomical (Gilbert and Wiesel, 1989; Sincich and Blasdel, 2001), psychophysical (Field et al., 1993; Kovács and Julesz, 1993; Polat and Sagi, 1993, 1994; Alais and Lorenceau, 2002; Hess et al., 2003; Cass and Alais, 2006), and modeling (review in Seriès et al., 2002) approaches converge to suggest that long-range horizontal cortical connections in primary visual cortex embed a sophisticated mechanism for processing visual contours, reminiscent of the principle of good continuation proposed by the Gestalt psychology (Koffka, 1935; Kellman and Shipley, 1991; Chavane et al., 2000). Anatomically, intrinsic long-range lateral connections extend over large distances (up to 8 mn, Gilbert and Wiesel, 1983; Fitzpatrick, 1996; Schmidt et al., 1997; Cavanaugh et al., 2002; Levitt and Lund, 2002) and link cells with non-overlapping receptive fields, predominantly neurons belonging to iso-orientation columns (Gilbert and Wiesel, 1979, 1983; Malach et al., 1993; Bosking et al., 1997; Levitt and Lund, 1997; Sincich and Blasdel, 2001). Horizontal connections are inhibitory as well as excitatory (Hirsch and Gilbert, 1991), although the majority of the postsynaptic effects of long-range intra-cortical interactions are excitatory (Nelson and Frost, 1985; Kisvarday et al., 1986; McGuire et al., 1991; Kapadia et al., 1999; Polat et al., 1998). In V1, stimulation in a cell's receptive field and in its surround shows that response modulation by remote stimuli depends on contrast and distance (Kapadia et al., 1995, 2000; Polat et al., 1998). Stimulation away from the receptive field of a recorded neuron does not elicit spiking activity in itself and only modulates the resting membrane potential (Nelson and Frost, 1985; Ts'o et al., 1986; Kapadia et al., 1995; Polat et al., 1998). Neural facilitation is observed with low-contrast stimuli while suppression dominates at high contrasts (Kapadia et al., 1999). Suppression or weak facilitation has been reported for non-collinear arrangements. Moreover, neurons tuned to the same orientation but with distant or not aligned receptive fields do not facilitate each other (Kapadia et al., 1995, 2000; Polat et al., 1998; Ito and Gilbert, 1999).

These converging anatomical and electrophysiological studies suggest that long-range horizontal connections underlie the integration of elongated contours (Field et al., 1993; Polat and Sagi, 1993, 1994; Polat et al., 1998; Kapadia et al., 1999, 2000) and that “association fields” (Field et al., 1993) are the basis of an inference process whereby a neuron activated by its preferred orientation propagates its “belief” that neighboring neurons should also be co-activated, given the statistical distribution of orientations in natural images (Geisler et al., 2001).

Most investigations of the temporal dynamics of contour coding (Usher and Donnelly, 1998; Beaudot, 2002; Polat and Sagi, 2006) varied the temporal asynchrony between co-aligned target element and randomly oriented non-target elements, with discrepant results. Usher and Donnelly (1998) reported enhanced contour detection for asynchronous target and background, but Beaudot (2002) did not replicate this finding and suggests it results from a strong priming effect when the target is presented first. However both studies used high contrast stimuli together with a path detection task where subjects have to report the presence or the position of several co-aligned elements. Polat and Sagi (2006) measured the detection threshold for a Gabor target flanked by high contrast Gabors while varying the temporal offset between the target and the flankers. The temporal asymmetry they report is interpreted as reflecting the different speed of excitation and inhibition. However, the use of double flash presentations—target followed or preceded by distractors—and high contrast stimuli may not be best suited to test the intrinsic dynamics of long-range interactions, as they mainly reflect interactions between a masking background and a target rather than estimate the flow of activity along long-range connections. Cass and Alais (2006) used a clever psychophysical paradigm where subjects must detect a static target Gabor flashed with varying time offset while flanker Gabors rotate over time. Plotting the contrast threshold as a function of phase lag they derive an optimal facilitative delay for target-flanker interactions commensurable with electrophysiological estimates of the propagation speed through long-range interactions.

The aim of the magnetoencephalography (MEG) experiment detailed below is twofold: (1) to investigate whether waves of activity modulate the latency and amplitude of MEG recordings in humans and (2) to test whether these hypothetical modulations depend on contrast and orientation, as would be expected if the network of long-range horizontal connections is involved.

In order to probe the effects of wave propagation on the amplitude and latency of cortical responses, it is desirable that the temporal course of the visual stimulation closely matches their cortical speed so as to maximize the interactions between lateral and feedforward inputs. Moreover, the stimulation should remain in a reasonable range to ensure it can be perceptually processed. To convert cortical speed into motion in visual space, one must take into account the magnification factor, M, which varies with eccentricity and between species (Seriès et al., 2002). Taking an estimate of M between 2 and 5 mm/deg, corresponding to an eccentricity of 2–6° for human retino-cortical projection (Dow et al., 1981; Sereno et al., 1995), leads to visual speeds ranging between 20 and 500°/s. Although this speed range is wide, it is worth noting that slow cortical waves correspond to very fast motion in visual space, at least much faster than usual ecological motion encountered in natural vision.

Electrophysiological recordings (Bringuier et al., 1999; Frégnac et al., 2010), psychophysical (Georges et al., 2002) and modeling (Seriès et al., 2002) studies suggest that when two co-aligned Gabor patches are flashed sequentially in two neighboring locations, the lateral activity propagated by the first patch can facilitate the response to the second patch provided that the temporal and spatial separations as well as the relative orientation between both Gabors are appropriately set. As this sequential stimulation also elicits a percept of apparent motion we shall refer to motion stimulation in the following. We relied on these previous studies to choose the speed, orientation and contrast of fast apparent motion sequences presented left or right from a fixation cross and examined whether the amplitude and the latency of MEG responses depend on propagating activity within the network of horizontal long-range connections.

**Figure 1:**
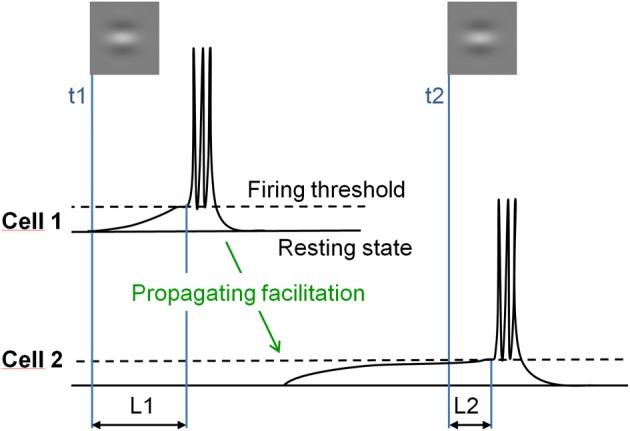
**Illustration of the hypothesis.** Responses of two orientation selective V1 neurons with co-aligned non-overlapping receptive fields stimulated in succession. Cell 1, stimulated first at time t1, depolarizes its neighbors through long-range horizontal connections with slow propagation speed (green arrow). Stimulation of the depolarized cell 2 at time t2 results in shortened response latency (L2), as compared to the response latency of cell 1 to the first input (L1).

Specifically, we tested whether spreading facilitation elicits a shortening of the response latency to incoming inputs. In the model of Seriès et al. (2002), long-range facilitation, propagating slowly through thin non-myelinited axons running horizontal to the cortical surface, depolarizes neurons' membrane potential after a delay. As a consequence, whenever a neuron is activated, it sends facilitating signals to neighboring neurons with similar orientation preference and receptive fields co-aligned in visual space (Figure 1).

This depolarizing wave, although it does not produce spiking responses in itself, reduces the time-to-firing threshold of neurons to subsequent feed-forward inputs, and thus their firing latency. This effect of depolarization on response latency increases with decreased contrast because time-to-threshold is longer at a low than at a high contrast. Crucially, the model predicts that low-contrast co-aligned Gabor patches presented in succession elicit neuronal responses with a shorter delay than misaligned Gabor patches. This effect should occur whenever lateral and feed-forward inputs temporally coincide, hence when successive stimulations are separated by short time intervals, and should decrease at high contrast because time-to-threshold is too short to be significantly modulated by lateral inputs in this case.

In the following, we test these predictions by analyzing the MEG response of humans passively viewing low or high contrast Gabor patches briefly flashed in successive positions along the vertical axis, depending on whether these patches are parallel or orthogonal to the vertical axis.

## Methods and protocol

### Participants

Ten volunteers (five men and five women), aged 22–28, participated in the study after giving their informed consent, and received financial compensation for their participation. All were right-handed, had a normal vision without correction and were naive with respect to the stimuli. Four had a left ocular dominance.

### Stimulation

**Figure 2:**
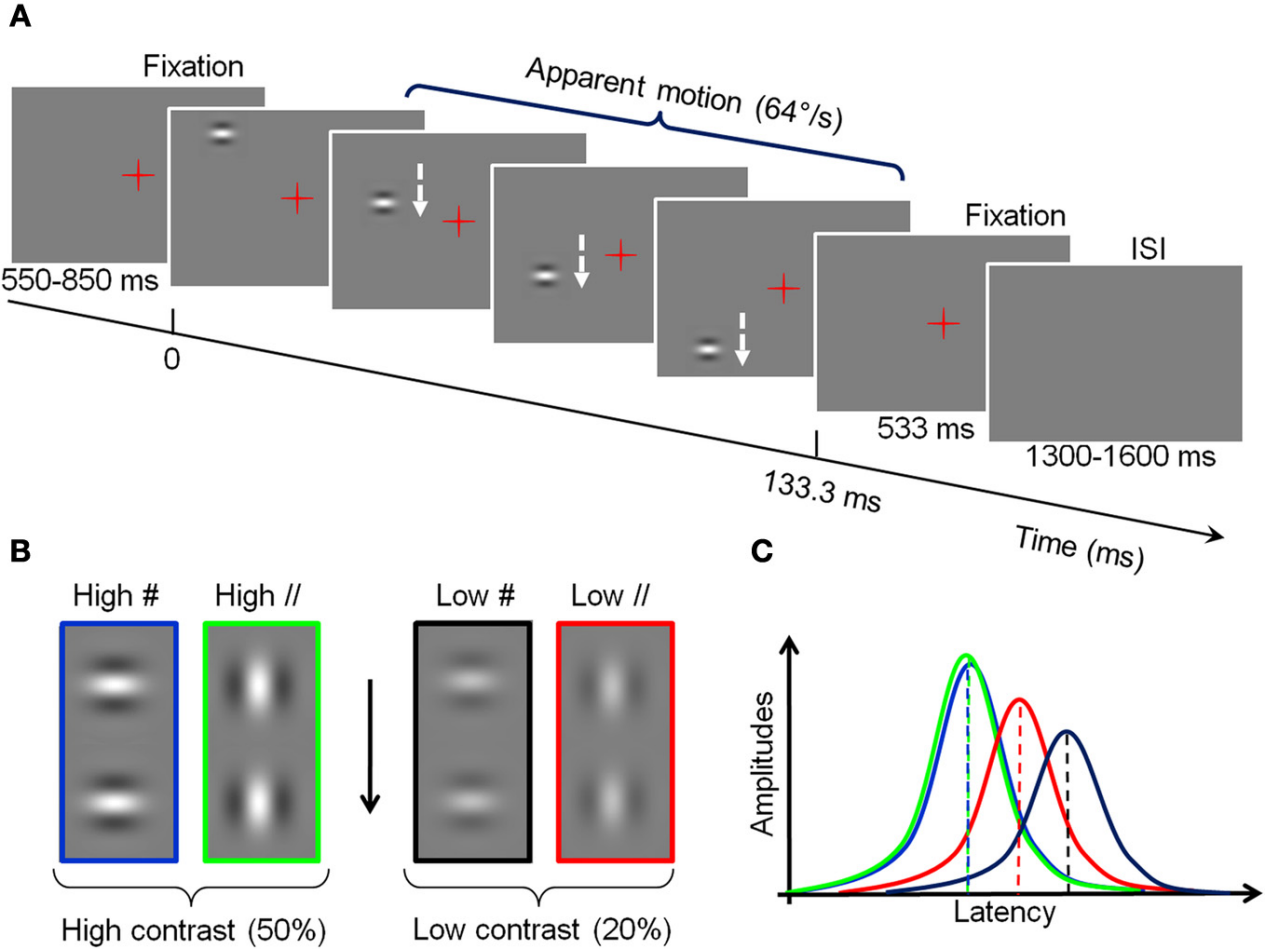
**Experimental design and hypotheses. (A)** Timing of a trial. A Gabor patch is presented sequentially in different positions along a vertical axis, left or right from fixation **(B)** High and low-contrast Gabor at two orientations used in the experiment. For visualization purpose, the contrasts of the figure do not correspond to the real ones. **(C)** Schematic representation of the effect of traveling waves on the amplitude and latency of cortical responses to the different conditions of stimulation. Green: High-contrast Gabor parallel to the vertical axis (High //). Blue: High-contrast Gabor orthogonal to the vertical axis (High #). Red: Low contrast, parallel (Low //). Black: Low contrast, orthogonal (Low #).

The stimuli consisted of Gabor patches displayed in sequence along a vertical axis located 2 degrees right or left from a central fixation, and inducing a fast apparent downward motion at 64°/s. In each trial, two fixation periods flanked this sequence (see Figure 2A). Each patch subtended 1.6° of visual angle (dva thereafter). Their mean luminance was the same as the background (189 Cd/m^2^) but was modulated horizontally or vertically with a spatial frequency of 1.5 cycle per degree (cpd), and their contrast followed a Gaussian profile (σ = 0.32 dva; see Polat and Sagi, 1993). The Gabor patches were either orthogonal to the vertical apparent motion path (#) or parallel to it (//). Depending on the conditions, the maximal Michelson contrast of the patches was 50% (“High” contrast) or 20% (“Low” contrast, see Figure 2B). These values are derived from our previous psychophysical study (Georges et al., 2002), chosen to be largely above threshold while entailing significantly different neural response latencies (as indicated in electrophysiological studies, e.g., Gawne et al., 1996). Stimuli were back projected on a screen, via a mirror system, using a calibrated Mitsubishi X120 projector (60 Hz refresh rate) located outside the shielded room. Subjects underwent seven blocks of 6 min, each comprising 128 trials, corresponding to 16 repetitions of the four conditions in each hemi-field, for a total of 112 trials per condition. The order of presentation of the conditions was randomized across blocks.

Subjects were given no other task than to stare at the fixation point and to avoid moving their head or blinking as long as the fixation point was on the screen. The ISI was long enough (random duration between 1300 and 1600 ms) to allow blinking during this period.

### MEG data

**Figure 3:**
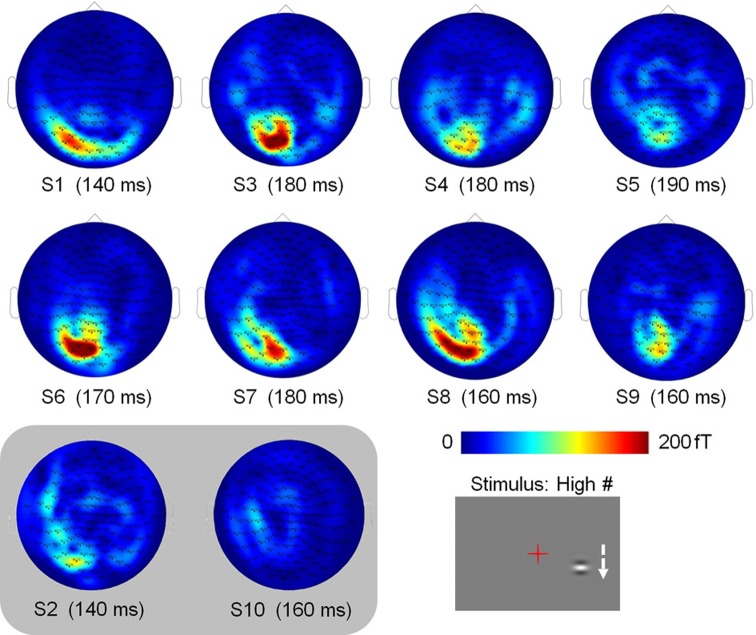
**Individual subject sensor topographies of the first peak of activity elicited by the High # condition, for the right stimulation (times indicated below).** Activity is consistently located in the left hemisphere. These topographies served to select the sensors eliciting maximum activity that are used in the analyses. Topographies of subjects S2 and S10, which were discarded from the analyses, are shown in a shaded inset.

Data were collected at the MEG-EEG centre (Paris, France) using a 151-channel whole-head system (3rd order radial gradiometers, CTF System, Port Coquitlam, British Columbia, Canada). Each stimulation block was continuously recorded with a sampling rate of 1250 Hz. The subjects' head position relative to the MEG sensors was controlled before each run. Horizontal and vertical eye movements were monitored with two pairs of surface electrodes (electro-oculogram EOG). Electrocardiogram (ECG) was also recorded. Trials contaminated with muscle artifacts, eye movements or blinks occurring between −540 and 460 ms around the stimulus onset were rejected on visual inspection. Overall, between 13 and 21% of the trials were discarded from the analyses. Importantly, the number of retained trials did not change across conditions (Friedman test: *p* > 0.95) and thus cannot account for possible differences. Planar gradiometer data were estimated from axial gradiometer data, by computing a linear interpolation between each sensor site and its closest neighbors. Such transformation has the advantage of giving topographies with maxima above the underlying cerebral sources; making them easier to interpret in particular with respect to the presentation side. Trials were low-pass filtered at 40 Hz, to filter out high–frequency noise and possible artifacts caused by electric power supply (50 Hz), and averaged with respect to stimulus onset, for each subject, each condition and each hemi-field of presentation. Data from two subjects were discarded because of low signal to noise ratio: Subject 2 presented high alpha activity in both the fixation and Gabor presentation periods (S2 in Figure 3), and subject 10 had the smallest amplitude of signal, with a maximal amplitude of signal in the reference condition (High #, see Figure 2) smaller than half the value of all other subjects for the right presentation (S10 in Figure 3).

### Data analysis

As stronger activity is expected for high-contrast stimuli, we chose the condition with high-contrast patches orthogonal to the vertical axis (High #) as the condition of reference to select the sensors of interest on an individual basis. For this condition, we found the time of maximal activity in the range of 50–250 ms and selected the 2 or 3 sensors with maximal activity at this peak (see topographies of the peak activity for both hemi-fields of presentation in Figure 3).

**Figure 4:**
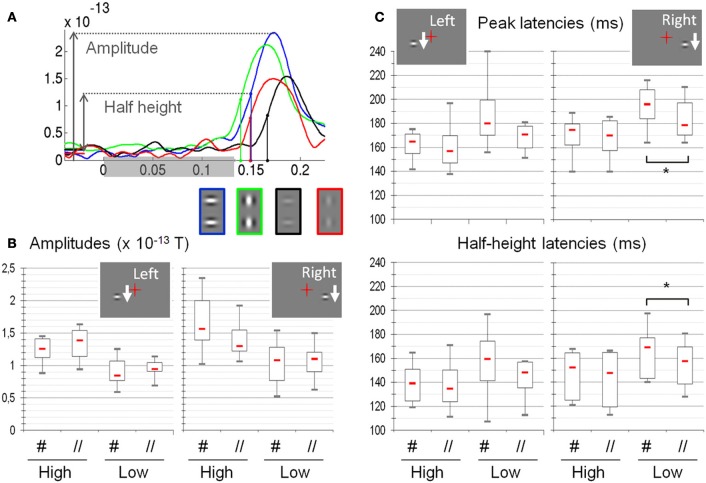
**Amplitude, latency and half-height latency measured on the MEG signals as a function of the different experimental conditions (*N* = 8). (A)** Example of individual time courses (subject S6, responses to right presentation conditions) averaged over selected sensors. The color of the curve codes for the experimental condition, reminded below (green: High //, blue: High #, red: Low //, black: Low #). The shaded rectangle on the horizontal axis shows the whole period of stimulation. **(B)** Amplitudes: High-contrast stimuli elicit larger responses than low-contrast stimuli. Stimulus orientation does not significantly modulate the response amplitude. **(C)** Latencies: The peak latencies and the half-height latencies are shorter for high as compared to low-contrast stimuli. At low contrast, response latencies are shorter for the parallel as compared to the orthogonal orientation, although this difference is significant only for right presentation (asterisks). Red dash: median values; box: interquartile range; whiskers: full range. See text for details.

The signal of the selected sensors was then averaged separately for each subject and condition. From these averaged signals, we extracted the peak value (amplitude), the peak latency and half-height latency (half height being the mean of the peak and a baseline value estimated between −120 and −20 ms, see Figure 4).

For these three measures, we performed repeated-measure ANOVAS with factors Contrast (High, Low) and Orientation (#, //), separately for each hemi-field of presentation. According to our hypotheses, a main effect of contrast and an interaction between contrast and orientation are expected.

Because of the limited number of subjects, non-parametric statistics were also performed (Friedman test, Georgin and Gouet, 2000). Wilcoxon tests were used to separately assess the main effects of Contrast (mean of high-contrast conditions vs. mean of low-contrast conditions) and Orientation (mean of # conditions vs. mean of // conditions) and the crossed effect of Contrast × Orientation (difference between High # and High // vs. difference between Low # and Low //). To better characterize a possible interaction, we also tested the orientation effect separately for the high contrast and low contrast conditions.

## Results

All results, computed from eight datasets are shown in Figure 4. The Friedman tests reveal that the four experimental conditions induce significant variations of amplitude (Right: *p* = 1.3 × 10^−4^ and Left: *p* = 4.1 × 10^−4^), peak latency (Right: *p* = 1.1 × 10^−4^; Left: *p* = 1.92 × 10^−3^) and half-height latency (Right: *p* = 1.5 × 10^−4^; Left: *p* = 1.02 × 10^−2^). According to *post-hoc* tests, the Low # condition always significantly differs from the High # condition (*p* < 0.05 corrected for multiple comparisons, Siegel and Andersen, 1988).

The ANOVAs (see Table 1) reveal a strong effect of contrast on the three measures (amplitude: *p* = 5.3 × 10^−5^ and *p* = 0.0019 for right and left presentations respectively; latency: *p* = 7 × 10^−5^ and *p* = 1.8 × 10^−5^; half-height latency: *p* = 9 × 10^−4^ and 0.0089). For right presentations, we also find a main effect of Orientation on the response latency (*p* = 0.049 for peak latency and *p* = 0.012 for half-height latency); and a significant interaction between Orientation and Contrast for both the amplitude (*p* = 0.0035) and the half-height latency (*p* = 0.019). However, we found no effect of orientation on the amplitude (*p* = 0.33) and no Orientation × Contrast interaction on peak latency (*p* = 0.11).

**Table 1:** **Results of the repeated-measure ANOVAs performed with factors Contrast (High and Low) and Orientation (/ and #) on Amplitudes, Latencies, and Half-height latencies**.

	**Left**			**Right**		
	***F***	***p***	**Partial eta^2^**	***F***	***p***	**Partial eta^2^**
**AMPLITUDE**						
Contrast	**23.19**	**0.0019**	**0.77**	**75.97**	**0.000053**	**0.91**
Orientation	0.68	0.44	0.09	1.05	0.34	0.13
Contrast × Orientation	0.20	0.67	0.03	**6.82**	**0.035**	**0.49**
**LATENCY**						
Contrast	**105.56**	**0.000018**	**0.94**	**69.35**	**0.00007**	**0.91**
Orientation	3.36	0.11	0.32	**5.67**	**0.049**	**0.45**
Contrast × Orientation	1.96	0.203	0.22	3.20	0.12	0.31
**HALF-HEIGHT LATENCY**						
Contrast	**12.83**	**0.0089**	**0.65**	**30.23**	**0.0009**	**0.812**
Orientation	2.54	0.15	0.27	**11.26**	**0.012**	**0.62**
Contrast × Orientation	1.09	0.33	0.13	**9.12**	**0.019**	**0.57**

*Significant effects (p < 0.05) are highlighted in bold*.

For left presentations, despite a clear effect of contrast, we find no effect of orientation either on the amplitude (*p* = 0.44) or on the latency of the responses (*p* = 0.1 and *p* = 0.15 for peak and half-height latency respectively). No interaction is found either (amplitude: *p* = 0.66; peak latency: *p* = 0.2; half-height latency: *p* = 0.33).

These results were systematically confirmed by non-parametric statistics (see Table 2). Wilcoxon tests on the data averaged by orientation confirm a main effect of the contrast on amplitude (*p* = 0.0059, one tailed test), latency (*p* = 0.0059) and half-height latency (Right: *p* = 0.0059 and Left: *p* = 0.0086). Wilcoxon tests on the data averaged by contrast show a main effect of the patch orientation on the latencies only for right presentations (Right: peak latency *p* = 0.009; half-height latency *p* = 0.0086; Left: peak latency *p* > 0.04; half-height latency *p* > 0.1; Both: amplitude *p* > 0.16; one-tailed tests). Finally, Wilcoxon tests on the differences between orientations confirm a significant interaction (orientation × contrast) for both the amplitude (*p* = 0.0191) and the half-height latency (*p* = 0.0125), for right presentation only (other comparisons *p* > 0.1, one-tailed tests).

**Table 2:** **Results of the non-parametric (Wilcoxon) tests performed on Amplitudes, Latencies, and Half-height latencies**.

	**Left**		**Right**	
	***p***	**Effect size**	***p***	**Effect size**
**AMPLITUDE**				
Contrast	**0.0059**	**33% (0.37 × 10^−13^*T*)**	**0.0059**	**37% (0.47 × 10^−13^*T*)**
Orientation	>0.16	5%	>0.16	5%
Interaction	>0.2	3%	**0.0191**	**21% (0.28 × 10^−13^*T*)**
**LATENCY**				
Contrast	**0.0059**	**15 ms**	**0.0059**	**20 ms**
Orientation	>0.04	7 ms	**0.009**	**6 ms**
Interaction	>0.1	10 ms	>0.1	8 ms
**HALF-HEIGHT LATENCY**				
Contrast	**0.0086**	**15 ms**	**0.0059**	**15 ms**
Orientation	>0.1	5 ms	**0.0086**	**7 ms**
Interaction	>0.2	6 ms	**0.0125**	**6 ms**

*Data were averaged across Orientation to test the main effect of Contrat and reciprocally. The interaction effect was tested by comparing the differences related to Orientation depending on Constrast. Significant effects (one-tailed *p* < 0.025) are highlighted in bold. Effect size are given in percentage of signal for the amplitude (the absolute amplitude of the effect is also given in Tesla for the significant effects) and in milliseconds for the latencies*.

The difference of peak amplitude between high and low-contrast stimuli is 35% (0.47 × 10^−13^ *T*) for right presentations and 33% (0.37 × 10^−13^ *T*) for left presentations. The latency difference is about 15 ms (right presentations: 20 ms for peak latency and 15.3 ms for half-height latency; left presentations: 15.2 ms latency and 14.95 ms for half-height latency).

The main effect of orientation on response latencies to right presentations corresponds to a phase advance of about 7 ms for parallel (//) as compared to orthogonal (#) patches (6.4 ms for peak latency and 7 ms for half-height latency, both significant). Note that for the left presentations, the mean delay between # and // is 7 ms for the peak latency and 4 ms for half-height latency, but these difference are not found significant.

The significant interaction between contrast and orientation for right presentation corresponds to a time difference of 8 ms for peak latency and 6 ms for half-height latency. For left presentations, we find the same amplitudes of effect on latency (10 ms) and half-height-latency (6 ms), but those are not significant.

When detailing the orientation effect, we observe that, at low contrast it corresponds to an average advance of about 10 ms for // with respect to # (Latencies: 10 ms for right presentations and 12 ms for left presentation; half-height latencies: 9 ms for both left and right), the difference being significant for right presentations only (Right: latencies, *p* < 0.006; half-height latencies, *p* = 0.009; Left: latencies *p* > 0.04; half-height latency: *p* > 0.011). At high contrast, the advance is about 2 ms only for peak latencies (Right: 2.5 ms; Left: 2 ms) and 4 ms for half-height latency (both left and right), none of these differences being significant.

**Figure 5:**
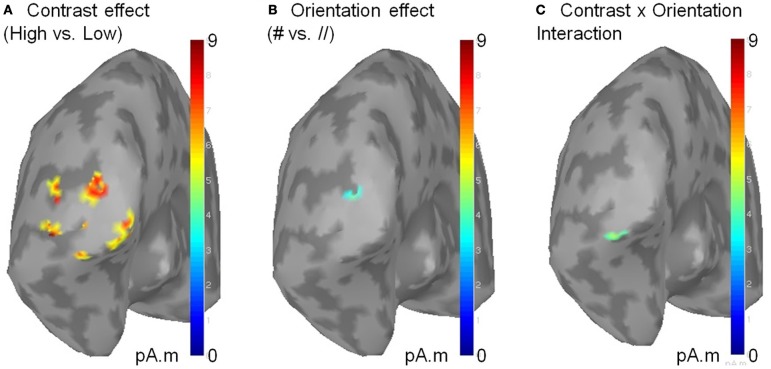
**Sources of the significant effects of contrast (A), orientation (B) and of their interaction (C).** Sources were reconstructed on the MNI template using a L2 minimum norm approach. Source activity was averaged on a 50 ms time window centered on the response peak of each subject before averaging across subjects. Only clusters of activity exceeding 51% of the maximal amplitude and extending on more than five vertices are displayed. As shown above, the contralateral sources of the contrast × orientation interaction are located within the occipital pole, presumably V1. No sources were found in the ipsilateral occipital cortex (not shown).

To gain information about the brain structures involved in these effects, we reconstructed the sources of the contrast and orientation effects as well as their interaction using a depth-weighted minimum L2 norm estimator of cortical current density applied on Collin MNI anatomy. MEG source analysis was performed using the BrainStorm software (http://neuroimage.usc.edu/brainstorm). The sources related to the effects of the parameters of interest were located in the occipital pole (Figure 5), suggesting that the observed effects mainly reflect activity in the primary visual cortex.

## Discussion

This study compared the magneto-encephalographic responses of humans passively viewing Gabor patches at different contrasts and orientations, displayed in fast sequences along a vertical axis, left or right from a static central fixation mark. High-contrast Gabors evoked responses in the occipital lobe that were both more ample (by ~30%) and occurred with shorter latencies (by ~15–20 ms), than low-contrast Gabor patches. The data further showed that low-contrast Gabors aligned with the vertical motion path elicited responses with shorter latencies than a Gabor orthogonal to the vertical axis (mean latency advance of ~7 ms). No such latency shifts were observed for high-contrast Gabors (~3 ms, non-significant).

The observed effects are unlikely to reflect a differential processing of vertical and horizontal orientation, as the results from previous psychophysical data were similar for a wide range of Gabor orientations. Although a control condition with different motion axes and Gabor orientation would be necessary to support this claim, a control condition using a horizontal motion axis would cause the stimulus to cross the vertical meridian and stimulate both hemispheres, which would entail difficulties for data analyses and valid comparisons between different motion axes.

The effects of contrast and orientation were similar in both hemispheres, but are only significant for visual motion sequences presented in the right hemi-field. We do yet not have a straightforward explanation of this left/right asymmetry that we also observed in a behavioral speed discrimination task with similar stimuli. We note that all subjects were right handed and can only speculate that the left/right difference could correlate with already known asymmetries in visual processing, as for instance pseudoneglect (Jewell and McCourt, 2000), or hemispheric differences in spatial frequencies and motion processing (Sergent, 1982; Peyrin et al., 2003) but further studies are needed to test these hypotheses.

Overall, the responses latencies reported here are relatively long as compared to a classical M100 response. The relatively late response latencies observed here for all conditions is likely to reflect the fact that the stimulus first enters the observers' visual field from the periphery and that interactions between elements can only occur after the occurrence of the second frame of a sequence.

The latency advance for high-contrast stimuli is in good agreement with previous electrophysiological recordings in macaque V1 (e.g., Gawne et al., 1996) and corroborates the effects of contrast and orientation found for intracellular recordings in anesthetized cat that also used fast Gabor sequences (Baudot et al., 2000; Lorenceau et al., 2001; Frégnac et al., 2010). A psychophysical speed discrimination study using stimuli similar to those used herein (Georges et al., 2002), reported that a Gabor patch aligned with the motion axis appears faster than a Gabor orthogonal to it. This speed-up effect is large at high speeds (PSE of 2 for 64°/s) and decreases at higher and lower physical speeds. Additional results (Paradis et al., 2011) found that a low-contrast Gabor (20%) aligned with the motion path is seen as moving much faster than a similarly oriented high-contrast Gabor (50%), an effect that disappears for Gabor patches orthogonal to the motion path. Altogether, these electrophysiological and psychophysical results suggest that the effects of contrast and orientation found with MEG reflect veridical and reliable processing differences.

The computational model developed by Seriès et al. (2002) accounts for the perceptual reports of Georges et al. (2002), and provides insights into the underlying mechanisms. The model relies on latency shifts mediated by long-range horizontal connections between cortical neurons responding in succession to oriented Gabors (Figure 1). In addition to simulating the psychophysical data, the model also predicts most of the effects of contrast and orientation reported here.

Crucially, the model predicts larger effects for a low-contrast Gabor aligned with the motion path than for other contrast and orientation configurations. This prediction stems from the fact that whenever a depolarizing input arising from a neighboring neuron through long-range facilitation brings a neuron close to its spiking threshold, its time-to-threshold is shortened. This shortening is large for low-contrast stimuli because the depolarization needed to reach the spiking threshold at low contrast is slow (long time-to-threshold; Figure 1), but the latency gain is smaller for high-contrast stimuli because depolarization is fast and the time-to-threshold is already short in this case. The present findings support this processing scheme, as a response phase advance is only observed for a low-contrast Gabor aligned with the motion path. In this view, latency shifts occur because horizontal facilitation—and/or suppression—between cells selective to similar—or dissimilar—orientations modulate the neurons' resting potential. As a consequence, the time needed to integrate the energy of an incoming feedforward input is modulated by signals from distant cortical neurons.

### Possible role of latency shifts in visual processing

What could be the functional role of latency shifts in visual processing? The dependency on contrast, orientation and speed provides constraints on what these putative roles can be. The perceptual speed-up effect detailed in Georges et al. (2002) reaches a maximum at a high speed (~64°/s). We speculate that, except with rare exceptions, this speed is much faster than the average speeds usually encountered in a natural environment (Calow and Lappe, 2007). It therefore seems unlikely that latency shifts can play a significant role in visual motion analysis. As saccadic eye-movements can induce very high retinal speeds, could latency shifts play a role in eye-movement control, for instance by speeding-up saccades or guiding eye movements toward relevant targets? The fact that saccades are mostly generated by sub-cortical structures such as the superior colliculus (Robinson, 1972; Wurtz and Albano, 1980), where neurons lack orientation selectivity, weakens the plausibility of this interpretation. An alternative is that latency shifts play no role in motion processing or eye-movement control and that the apparent motion paradigm used herein was only appropriate to temporally decompose the neural dynamics that would otherwise had gone unnoticed with stationary stimuli, because they are below perceptual thresholds. In this view, the latency shifts induced by spatial facilitation could reflect the propagation of “beliefs” between neurons, based on the high probability that neighboring neurons sharing similar orientation preference are stimulated by a single elongated contour (Geisler et al., 2001; Geisler, 2008), and serve to help contour extraction in cluttered environments.

**Figure 6:**
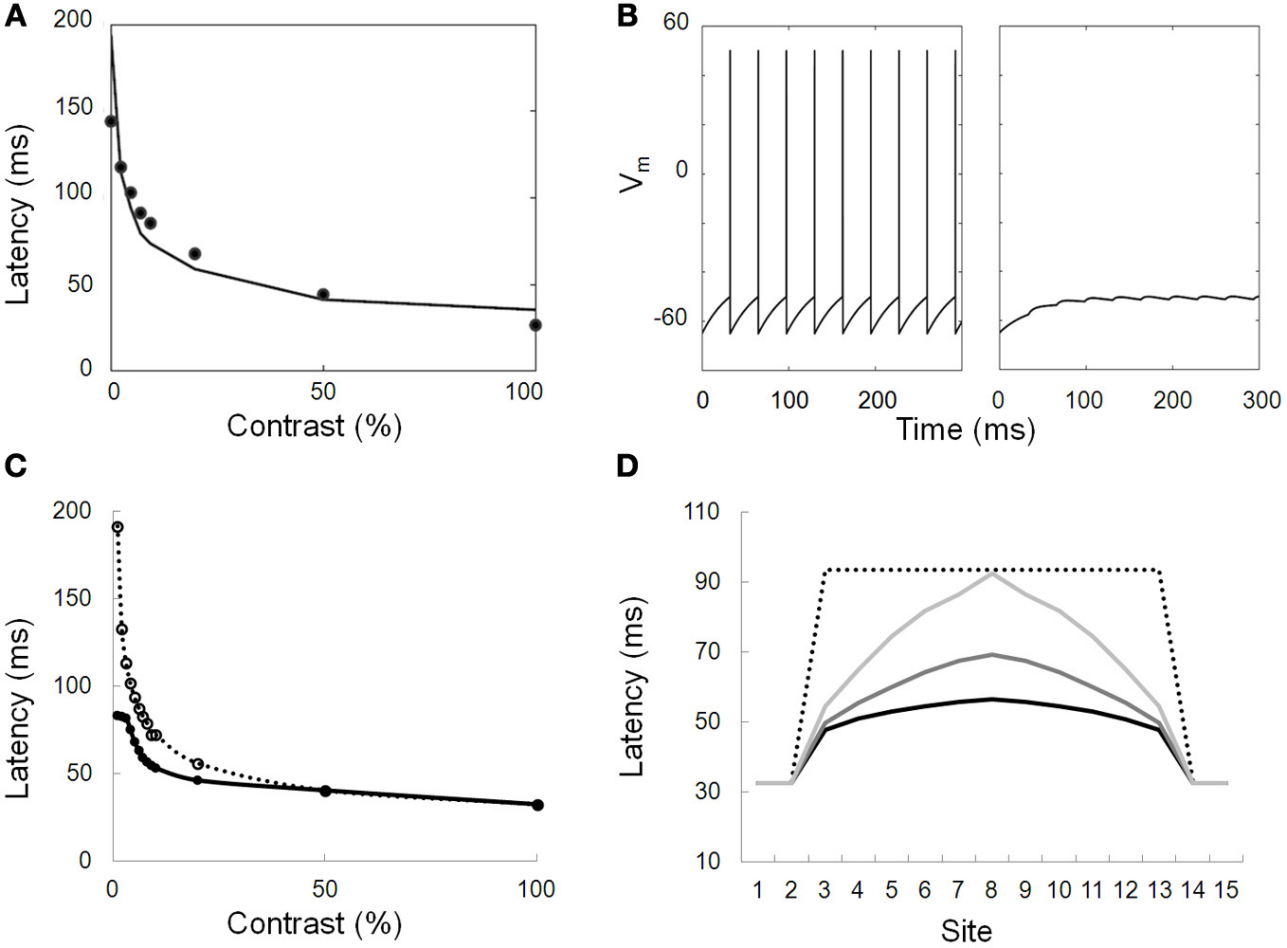
**Modeled responses of neurons. (A)** Simulation of the contrast latency (first spike) response function. Dots: data from electrophysiological recordings in macaque V1 (Gawne et al., 1996). Solid line: modeled function that provides a good fit to the data. **(B)** Modeled response of a neuron directly stimulated by a 100% contrast stimulus (left) and of a neighboring neuron only receiving lateral inputs from the first cell through long range connections. The strength of the lateral inputs is chosen so that only subthreshold depolarizing responses are elicited. **(C)** Simulated response latencies of a neuron either connected to (solid line, closed symbols) or disconnected from (dotted line, open symbols) its neighbor. The simulated neuron is activated by different input contrasts while its neighbor is stimulated with a high-contrast stimulus. At low contrast, the response latency is decreased by as much as 100 ms. **(D)** Simulated response latencies of 15 aligned neurons presented with a step of decreased contrast (only neurons 1,2,14, and 15 are presented with a maximal contrast). The profile of response is displayed for disconnected neurons (dotted line) and for neurons connected with different propagation delays (1, 4, and 10 ms corresponding to light gray, middle gray and black solid lines, respectively).

Considering natural contours provides arguments in favor of this view: In natural images, an elongated contour rarely has a homogenous contrast, owing to the background against which the contour is presented, the illuminant or the presence of occluding objects, as exemplified in Figures 6A,B where regions with high and low contrasts define a single elongated contour. One consequence of this contrast heterogeneity is that neurons responding to different local contrasts should do so with different latencies. Therefore, response latencies should be scattered in time and the variance of their distribution should be high. High spike time variance would in turn limit the efficacy of feed-forward inputs that drive neurons at subsequent processing stages (Azouz and Gray, 2000). We propose that one of the consequences of long-range collinear facilitation is to reduce spike time variance by temporally aligning the slower responses to low-contrast regions with the earlier fast responses to high-contrast regions.

According to the present findings and the model predictions, one consequence of the observed latency shifts is that neurons stimulated by a single elongated contour with varying contrast tend to align their responses in time, thus providing a simple and efficient spike time alignment mechanism (STAM) that synchronizes the neuronal activity tied to a figure contour, hence reducing the inherent latency scattering due to the varying local luminance and contrast of an input image. Moreover, a selective latency advance of the neural responses to collinear alignments, relative to non-aligned –e.g., background- elements could provide a temporal segregation of the responses to the contour figure from the responses to background contrasts (especially if long-range inhibition between cells with non-aligned receptive fields hyperpolarize their membrane potential, yielding longer time-to-threshold). The latency advance of a more synchronized population of neurons responding to a single contour in primary visual cortex could in turn entail earlier and stronger responses in higher visual areas –e.g., V2, MT—where a similar synchronizing mechanism could also exist, owing to the specific long range connectivity in these areas endowed with particular functional properties, as movement direction in MT. The feedback responses from these high level areas onto lower areas could further enhance the synchronicity and phase advance of their neural targets. Such a mechanism would provide a powerful way of selectively synchronizing neurons recruited by contour structures within images, endowing them with a temporal advantage relative to neurons responding to non-contour features.

### Spike time alignment model

To test STAM, we developed a new variant of the computational model of Seriès et al. (2002) and probed its responses to static elongated contours with contrast varying along the contours (Figures 6A,B). Each neuron is described using a conductance-based integrate-and-fire model whose response latency decreases with increasing contrast in a way similar to electrophysiological data (e.g., Gawne et al., 1996; Figure 6A). Neurons with similar orientation preference and co-aligned receptive fields are connected by long-range horizontal connections. The lateral connections are excitatory and, although they are unable to drive a firing response, depolarize their target neurons with a delay, as shown in Figure 6B. This delay represents a combination of the speed of propagation through long-range connections and of the distance between connected units^1^.

We then tested the model with artificial and natural contrast distributions and measured the neuronal response latencies with varying propagation delays as a function of the connection strength. We then compared these distributions with a network devoid of lateral connections (connection strength set to zero).

**Figure 7:**
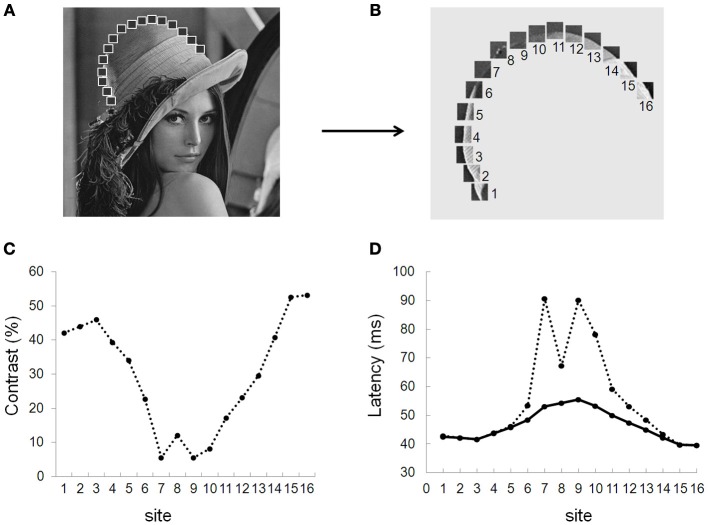
**Spike time alignment model (STAM).** Simulated distribution of response latencies of the STAM model for a series of patches at different contrasts drawn from the picture of Lena **(A,B)**. **(C)** Distribution of contrast along Lena's hat border for all sites (1–16). **(D)** Response latencies from the modeled neurons with (solid line) and without (dotted line) long-range connections. In this simulation, a propagation delay of 1 ms entails a spike time alignment of neuronal responses. The resulting decrease in the standard deviation of spike time distribution (from 17.5 to 5.3 ms) increases the probability that neurons at further processing stages summate their inputs within their temporal integration window, augmenting the probability of firing spikes.

Our simulations demonstrate that long-range connections influence the distribution of response latencies to elongated contour with varying contrasts (Figures 6C,D). Crucial aspects that emerge from the simulation are the following: (1) Early responses to high-contrast regions shorten the response latency of neighboring neurons stimulated by lower contrast regions; (2) As a result, the variance of the latencies of the recruited population is reduced as compared to a similar network lacking long-range connectivity. In the simulation shown in Figure 7 with a propagation delay of 2 ms, the standard deviation of the spike time distribution drops from 17.5 ms without lateral connections to 5.3 ms with lateral connections.

The observed latency advances depend on the relative contrast between neighboring neurons. They are small (<2 ms) at high contrast but can increase up to 50 ms when the contrast difference is large (Figure 6D).

STAM is consistent with the data and model of Cheadle et al. (2008) showing that judgment of temporal synchrony between temporally modulated targets embedded within a smooth contour are impeded relative to similar targets embedded in a jagged contour. In Cheadle et al's model (Cheadle et al., 2008), synchronization of responses via lateral interactions alters the temporal relationships between responses to temporally modulated targets, thereby limiting the perceptual ability to use internal responses to perform temporal judgments. A prediction derived from STAM is that the effects reported by Cheadle et al. (2008) should increase at low relative to high contrast. The STAM model is also reminiscent of the computational model of Van Rullen et al. (2001) where lateral connections modify the responses to elongated contours, although the model privileges spike order and does not explicitly considers spike time variance as a relevant parameter for contour integration. In conclusion, STAM demonstrates the plausibility of the model derived from the experimental data collected in MEG with fast apparent motion sequences. STAM relies on well-known properties of cortical V1 responses to orientation and contrast. The parameters used to simulate long-range horizontal connections are derived from experimental recordings in cat and monkey and can be widely varied with qualitatively similar outcomes.

Although STAM relies on long-range horizontal connections in V1, alternative implementations are conceivable. In particular, feedback connections from higher visual areas (e.g., V2, MT), known to modulate neuronal activity in primary visual cortex (Hupé et al., 2001b; Angelucci and Bullier, 2003), could change the cells' membrane potential in a way similar to that implemented herein. One requirement, however, would be that these feedback connections are orientation specific and target neurons with similar orientation preference. In addition, one would expect that the strength or density of these feedback connections is anisotropic in order to account for the present results, namely the difference between parallel and orthogonal configurations. Although the reconstruction of sources of the present MEG results suggests the observed effects originate from V1, it is not possible at this stage to decipher whether feedback from higher visual areas or long-range horizontal connections or both underlie the latency shortening reported here for co-aligned low contrast elements. Further studies with different methodologies (e.g., optical imaging, multielectrode recordings) would be necessary to clarify this issue.

In the present study, the responses of the modeled neurons only depend on the contrast/latency response function and on the weight and propagation speed of the lateral inputs. Previous studies have shown that neuronal response latencies also depend on other dimensions (Bullier, 2001). For instance, low spatial frequency information is mostly processed by fast magnocellular neurons with short response latencies while high spatial frequency information is mostly processed by slower parvocellular neurons with longer latencies (Bullier, 2001). Extending STAM to simulate magno and parvocellular neurons with spatial-frequency dependent response latencies would implement an early “coarse-to-fine” facilitation of visual processing (Bar, 2003) where magnocellular neurons stimulated by extended contours embedding different spatial scales would facilitate parvocellular neurons with co-aligned receptive fields. In this view, STAM predicts that the spike time variance of the population response to different spatial scale patterns should be decreased when these different patterns belong to a same contour.

### Conflict of interest statement

The authors declare that the research was conducted in the absence of any commercial or financial relationships that could be construed as a potential conflict of interest.

## Footnotes

^1^So neither distance nor propagation speed is explicitly represented in the model.

## Appendix: model description

The model describes a network of *N* neurons in early visual cortex (e.g. V1), with non-overlapping receptive fields. Those *N* neurons are interconnected through lateral connections.

### Neuron model

Each neuron *i* is described using a conductance-based integrate-and-fire model:

(1) $$\tau_{\text{m}}\frac{dV_{i}}{dt}=E_{L}-V_{i}+R_{m}I_{i}^{S}+I_{i}^{lat}$$

where *E*_*L*_ = −65 mV, *R*_*m*_ = 40 M, τ_M_ = 30 msec. When the membrane potential reaches *V*_th_ = −50 mV, the neuron fires a spike and the potential is reset to *V*_reset_ = −65 mV. Is denotes the visual feed-forward input, while *I*_lat_ denotes the recurrent input (cf below).

### Stimulus input

*I*_*s*_ is a function of the contrast of the image patch shown in the receptive field,

(2) $$I_{i}^{S}=K.{\text{log}}_{10}(c_{i}+c_{0}.G(\theta -\theta_{i})+c_{1})$$

with *K* = 0.3, *c*_0_ = 7 and *c*_1_ = 10 and *c*_*i*_ in range [1, 100]. We assume that the neurons are stimulated with their preferred orientation: G(θ − θ_*i*_) = 1 if there is a stimulus in their receptive field, and 0 otherwise.

This model and the parameters of the neuron were chosen so that the latency response approximates experimental data in monkey visual cortex [1] (Figure 1).

The contrast of the image patches are computed using the standard deviation of the normalized luminance values:

(3) $$c_{i}=\sqrt{\frac{1}{MN}\sum_{k\;=\;1}^{N}\sum_{j\;=\;1}^{M}{\left(I_{kj}-<I>\right)}^{2}}$$

where < *I* > denotes the mean normalized luminance in the patch and where *I*_*kj*_ are the *k*_*th*_ − *j*_*th*_ element of the two dimensional image patch of size M × N shown in the receptive field.

### Lateral input

The neurons are connected via long-range horizontal connections and excitatory synapses.

(4) $$I_{i}^{lat}=-\sum_{j}r_{m}g_{ij}(V-E_{s})$$

with *E*_*s*_ = 0 mV. *P*_*ij*_ follows:

(5) $$\tau_{S}\frac{dP_{ij}}{dt}=-P_{ij}$$

making the replacement *P*_*ij*_ → 1 after each presynaptic action potential of neuron j and with a synaptic delay τ_ij_. τ_s_ is set to 5 ms. and the delay between two adjacent neuron is fixed to τ_ij_ = 2 msec.

Because each neuron is representative of a larger population of neurons sensitive to the same region of visual space, the amplitude of the PSPs is assumed to describe that of a compound EPSP. The value of g_ij_ is chosen so that long-range horizontal inputs are not strong enough to elicit a spike by themselves.

Synaptic weights are assumed to decrease linearly with distance, while delays increase linearly.
